# Fibromyalgia in obstructive sleep apnea-hypopnea syndrome: a systematic review and meta-analysis

**DOI:** 10.3389/fphys.2024.1394865

**Published:** 2024-05-20

**Authors:** Jie He, Meifeng Chen, Na Huang, Bo Wang

**Affiliations:** ^1^ Clinical Medical College of Chengdu Medical College, Chengdu, Sichuan, China; ^2^ Department of Pulmonary and Critical Care Medicine, The First Affiliated Hospital of Chengdu Medical College, Chengdu, Sichuan, China

**Keywords:** fibromyalgia, obstructive sleep apnea-hypopnea syndrome, meta-analysis, incidence, sleep indices

## Abstract

**Introduction:** Fibromyalgia (FM) is a common condition in patients with obstructive sleep apnea-hypopnea syndrome (OSAHS). This meta-analysis aimed to evaluate differences in sleep monitoring indicators between patients with OSAHS and positive FM and patients with OSAHS and negative FM and to determine the incidence of FM in patients with OSAHS.

**Methods:** An exhaustive literature review was conducted to analyze the incidence of FM in patients with OSAHS, using online databases, including PubMed, EMBASE, Web of Science, CNKI, and Wanfang, both in English and Chinese. The quality of the included studies was assessed by two researchers using the Newcastle−Ottawa Scale scores. The acquired data were analyzed using Stata 11.0 software. Continuous variables were combined and analyzed using the weighted mean difference as the effect size. Conjoint analyses were performed using random-effects (I^2^ > 50%) or fixed-effect (I^2^ ≤ 50%) models based on I^2^ values.

**Results:** Fourteen studies met the inclusion criteria. This study showed that 21% of patients with OSAHS experienced FM. Subgroup analyses were performed based on race, age, sex, body mass index, and diagnostic criteria for patients with OSAHS. These findings indicate that obese patients with OSAHS have a higher risk of FM, similar to females with OSAHS. Regarding most sleep monitoring indicators, there were no discernible differences between patients with OSAHS with positive FM and those with negative FM. However, patients with positive FM had marginally lower minimum arterial oxygen saturation levels than those with negative FM. The current literature suggests that patients with OSAHS have a high incidence of FM (21%), and FM has little effect on polysomnographic indicators of OSAHS.

**Systematic Review Registration:** https://www.crd.york.ac.uk/prospero/display_record.php?ID=CRD42024510786, identifier CRD42024510786

## 1 Introduction

Fibromyalgia (FM) is a rheumatic disorder characterized by non-articular rheumatic pain syndrome, atopic myalgia, and hyperalgesia, the diagnosis and treatment of which are poorly understood (Passos et al., 2020). In addition to chronic pain, joint tenderness, muscle fatigue, sleep problems, cognitive impairment, and depression may occur (Tesio et al., 2018). FM affects 2–4% of the population, 80–90% of whom are females, and the age of onset is between 30 and 50 years (Gardoki-Souto et al., 2022). Although the exact cause of FM is unknown, it is known to be due to increased pain sensitivity due to dysfunction of the central nervous system (Kiso et al., 2018). Genetic and environmental factors are associated with the development of FM. Studies have shown that individuals who may suffer from FM may be at increased risk if their first-degree relatives are affected (D'Agnelli et al., 2019; Altınbilek et al., 2019). Certain family environmental factors, including acquired coping mechanisms for life challenges, have been implicated as inherent components in the development of FM (Bergman, 2005).

Obstructive sleep apnea-hypopnea syndrome (OSAHS) is a disorder characterized by apnea, decreased respiration, and decreased oxygen saturation level due to collapse of the upper airway, manifested by daytime sleepiness, fatigue, and inattention (Pei and Gui, 2021). The signs and symptoms of OSAHS and FM are similar. Treatment of FM is ineffective, and patients with FM often experience sleep problems, fatigue, and pain (Oka et al., 2020; Pei and Gui, 2021). Moldofsky et al. originally defined FM syndrome as musculoskeletal pain, fatigue, and sleep disturbances (Moldofsky et al., 1975). Most studies have examined the sleep quality of patients with FM, as there is no discernible difference in sleep duration between patients with FM and healthy controls (Moldofsky, 1989; Shaver et al., 1997). Similar sleep patterns, feelings of restlessness, and daytime sleepiness were observed in both the OSAHS and FM groups, suggesting a possible correlation between the two disorders. Mutlu et al. (Mutlu et al., 2020) evaluated female patients with FM using polysomnography (PSG) based on the results of a daytime sleepiness questionnaire and found that 65.9% of the patients had concurrent OSAHS. Sepici et al. (Sepici et al., 2007) performed PSG in a 55-year-old woman with FM for up to 10 years after complaining of waking up in the morning with fatigue, restless sleep, and daytime sleepiness, indicating severe OSAHS. These findings suggest that sleep monitoring in patients with FM is highly warranted; consequently, the incidence of FM in patients with OSAHS warrants further investigation.

Meresh et al. (Meresh et al., 2019) conducted a retrospective single-center study and identified a potential relationship between OSAHS and FM. However, this study did not systematically analyze the incidence of FM in patients with OSAHS. Furthermore, the incidence of FM may vary between OSAHS subgroups. Therefore, to statistically evaluate the incidence of FM in OSAHS, generalization and meta-analysis of all available data are critical. We investigated and analyzed the pathophysiology of FM in patients with OSAHS, as well as the link between the two conditions. We compared the differences in sleep monitoring indicators between patients with OSAHS and positive FM and between those with OSAHS and negative FM.

## 2 Materials and methods

### 2.1 Literature search strategy

Our meta-analysis was registered in the Prospective Register of Systematic Reviews (PROSPERO, https://www.crd.york.ac.uk/PROSPERO/, ID: CRD42024510786). We conducted an exhaustive search of databases, including PubMed, Web of Science, Wanfang, CNKI, and EMBASE, for research on the start of FM in patients with OSAHS. The time restriction for the search was established from the inception of the database until 1 March 2024. The keywords and subject phrases included “fibromyalgia” or “fibromyalgia syndrome” and “obstructive sleep apnea-hypopnea syndrome” or “obstructive sleep apnea” or “OSA” or “OSAHS” or “OSAS.”

### 2.2 Inclusion and exclusion criteria

The conditions for eligibility are outlined below.

1) Cohort studies, cross-sectional studies, case-control studies, or randomized controlled trials.
2) FM diagnostic criteria: Participants must have a clinical diagnosis that satisfies the diagnostic criteria of FM established by the American College of Rheumatology (ACR) in 1990, 2010, and 2016 (Wolfe et al., 1990; Wolfe et al., 2010; Wolfe et al., 2016). There were no age restrictions for participation.
3) Based on PSG, subjects met OSAHS diagnostic criteria (adults: apnea-hypopnea index [AHI] score ≥5/h, child: AHI score ≥1/h) (Li and He, 2021).

Traditional criteria (adults: AHI score <5, normal; AHI score 5–14, mild OSAHS; AHI score 15–29, moderate OSAHS; and AHI score ≥30, severe OSAHS, child: AHI score <1, normal; AHI score 1–5, mild OSAHS; AHI score 5–9, moderate OSAHS; and AHI score ≥10, severe OSAHS) were used to determine the severity of OSAHS (Baker et al., 2017).

The following were the exclusion criteria:

1) Letters, reviews, editorials, case reports, and other types of literature review.
2) Unable to obtain sufficient information from the original article or contact the corresponding author for additional information.
3) Studies without human participants.
4) Studies involving patients with OSAHS who had cancer, endocrine disorders, cerebrovascular accidents, chronic heart failure, or chronic respiratory diseases in the past.
5) Overlaps between studies and data from the same author.

### 2.3 Literature selection

Two researchers conducted separate searches of the aforementioned databases for pertinent articles, following the aforementioned criteria. They screened potentially eligible articles by reviewing their titles and abstracts, procured complete texts, and meticulously reviewed the full texts for reevaluation. A third researcher examined the disagreement between the two researchers about the inclusion or exclusion of an article and consulted the data to decide on its inclusion or exclusion.

### 2.4 Quality assessment of literature

One author assessed the quality of the studies using the Newcastle−Ottawa Scale (NOS) (Stang, 2010). Higher and equal to six “stars” were considered of great quality; between three and five “stars” were considered of moderate quality; and less than or equal to two “stars” were considered of low quality.

### 2.5 Data extraction and management

Initially, two researchers retrieved and assessed the data separately from the literature in the following manner: (Passos et al., 2020): basic information about the article: first author, publication date, and country; (Tesio et al., 2018); number and incidence of patients with positive FM and OSAHS; (Gardoki-Souto et al., 2022); baseline parameters for comparison in the study population, such as body mass index (BMI), AHI scores, mean oxygen saturation level, minimum oxygen saturation level, total sleep time, and sleep monitoring indicators; (Kiso et al., 2018); measurements of OSAHS and their study types; and (D'Agnelli et al., 2019) study quality. The researcher contacted the authors by phone or email at least twice to determine if the included literature lacked the necessary information and if they agreed to provide the missing data.

### 2.6 Statistical analysis

Stata software (version 11.0; StataCorp LLC, College Station, TX, United States) was used to compile and analyze the retrieved data. With a 95% confidence interval (CI), we normalized and represented continuous variables as weighted mean differences (WMD). The statistical heterogeneity of the included studies was determined using the I^2^ statistic in the heterogeneity test. A fixed effects model was used for the analysis and *p* ≥ 0.10 and I^2^ < 50% indicated that there was no statistical heterogeneity between the studies. The random-effects model was used for analysis when *p* < 0.10 or I^2^ > 50%, suggesting that there was statistical heterogeneity between studies. Subgroup and meta-regression analyses were performed to explore the sources of heterogeneity. The entire population was divided into groups according to mean age, mean BMI, sex, diagnostic criteria, and ethnicity for the subgroup analysis. For the sensitivity analysis, which investigated how each study affected the overall effect size, one study was eliminated at a time. The publication bias of the included literature (≥10 articles) was examined using Begg’s and Egger’s tests on Stata 11.0.

## 3 Results

### 3.1 Publications retrieved and included in the study

**FIGURE 1:**
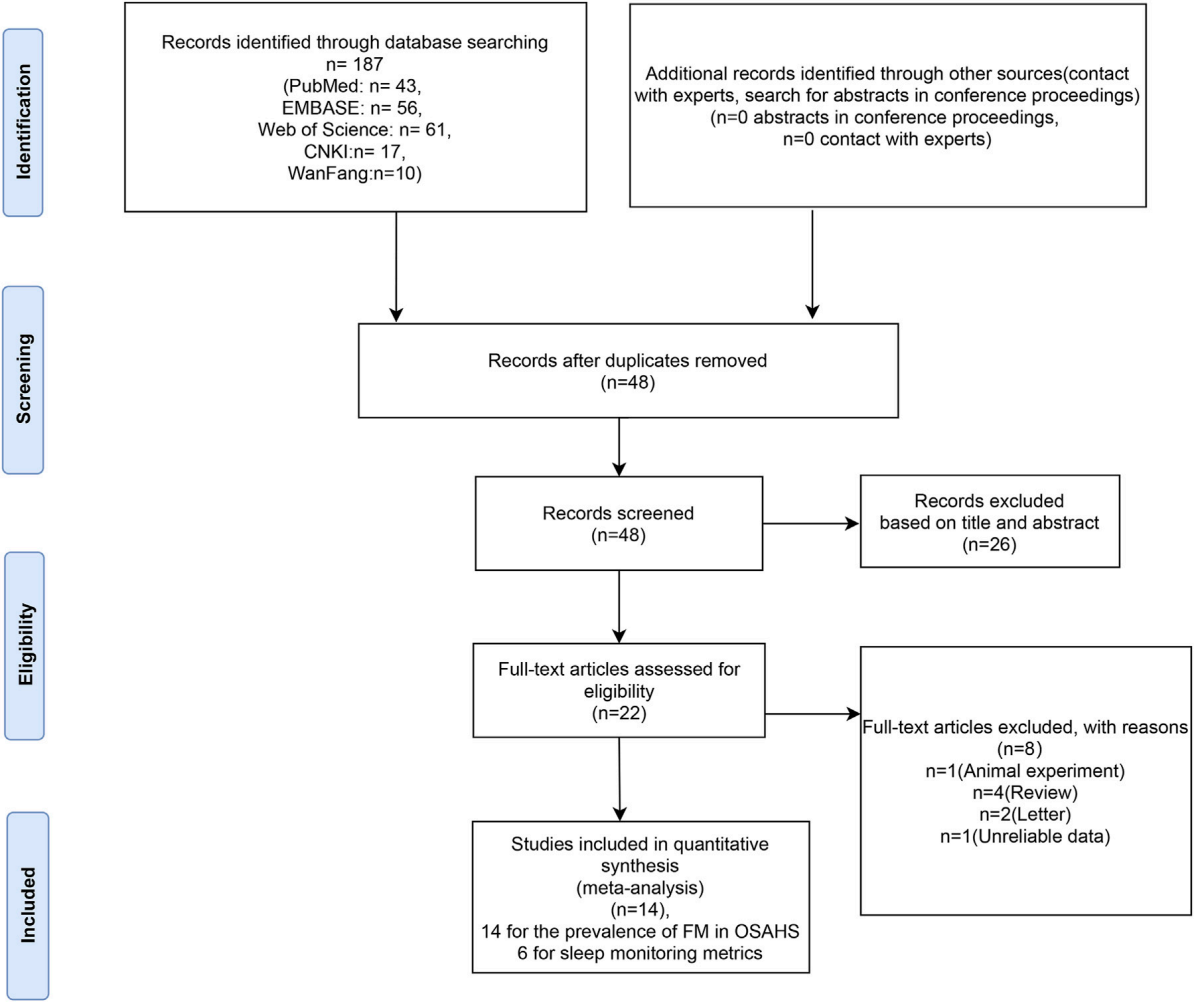
Flow diagram of literature screening.

A total of 187 relevant research articles were retrieved from the database. After screening the abstracts and titles, 139 duplicate studies were excluded and 48 were included. Twenty-six articles were excluded because they were irrelevant to the topic title. Eight of the 22 articles were eliminated after a comprehensive analysis of their entire texts and downloads, as well as a review of the inclusion and exclusion criteria. Four of these articles were reviews in which two were letters to the editor, one lacked pertinent data, and the other was an animal study. Therefore, these articles were excluded. Finally, 14 articles were included in our meta-analysis (Figure 1). The prevalence of FM in patients with OSAHS was reported in a total of 14 articles (Meresh et al., 2019; Dahan et al., 2006; O'Donovan et al., 2006; Wahner-Roedler et al., 2007; Chung et al., 2014; Rosenfeld et al., 2015; Terzi and Yılmaz, 2017; Yildirim and Alp, 2017; Köseoğlu et al., 2017; Johnson et al., 2020; Sanders et al., 2020; Wickwire et al., 2020; Altıntop Geçkil and Aydoğan Baykara, 2022; Cigdem Karacay et al., 2023). Six articles (Rosenfeld et al., 2015; Köseoğlu et al., 2017; Terzi and Yılmaz, 2017; Yildirim and Alp, 2017; Altıntop Geçkil and Aydoğan Baykara, 2022; Cigdem Karacay et al., 2023) compared sleep monitoring metrics in patients with positive FM and OSAHS to those with negative FM. The AHI scores, minimum SaO_2_ level, mean saturation oxygen level, total sleep duration, efficiency, latency, rapid eye movement (REM), N1, N2, N3, and Epworth sleepiness scale scores were among the markers used for sleep monitoring. Basic information on the included studies is presented in Table 1. Detailed sleep metrics are shown in Supplementary Tables S1, S2.

**TABLE 1:** Characteristics of included studies.

Author	Year	Country	Ethnicity	Age (year)	Total sample size	Patients with FM	Prevalence of FM	BMI (kg/m^2^)	Female/Male	NOS
Dahan V	2006	Canada	Caucasian	NA	60	16	0.267	27.70 ± 5.40	NA	6
O'Donovan CA	2006	United States	Caucasian	40.3 ± 5.5	26	5	0.190	28.8 ± 3.2	18\8	6
Wahner-Roedler DL (Male)	2007	United States	Caucasian	56.4 ± 10.2	267	6	0.022	27.4 ± 6.6	0\267	7
Wahner-Roedler DL (Female)	2007	United States	Caucasian	56.7 ± 11.0	139	22	0.158	26.9 ± 4.3	139\0	7
Chung DS	2014	Taiwan	Asian	47.10 ± 15.70	2,940	885	0.301	28.1 ± 4.5	1,145/1795	7
Rosenfeld VW	2015	United States	Caucasian	48.60 ± 11.10	385	133	0.345	30.1 ± 6.4	243\142	6
Terzi R	2017	Turkey	Caucasian	51.35 ± 8.68	31	6	0.194	33.55 ± 5.81	31/0	7
Yildirim T	2017	Turkey	Caucasian	50.60 ± 10.30	181	50	0.276	26.0 ± 4.8	57\74	8
Koseoglu Hi	2017	Turkey	Caucasian	51.75 ± 8.00	52	12	0.231	40.66 ± 11.53	16\36	8
Meresh ES	2019	United States	Mixed	53.27 ± 13.13	14,908	2,206	0.148	29.2 ± 3.2	5367\9541	8
Johnson KG (Female)	2020	United States	Caucasian	48.30 ± 13.50	195	27	0.138	35.50 ± 8.10	195\0	6
Johnson KG (Male)	2020	United States	Caucasian	49.50 ± 14.00	110	4	0.036	31.30 ± 6.90	0\110	6
Sanders AE	2020	United States	Caucasian	34.30 ± 1.10	793	52	0.065	28.17 ± 4.6	353\440	7
Wickwire EM	2020	United States	Mixed	72.50 ± 5.80	3229	817	0.253	27.2 ± 6.3	1540\1689	6
Altintop Geckil A	2022	Turkey	Caucasian	52.10 ± 11.90	88	41	0.463	35.10 ± 7.20	88\0	7
Cigdem Karacay B	2023	Turkey	Caucasian	55.40 ± 6.60	69	27	0.391	31.80 ± 5.00	17\10	8

FM, fibromyalgia; BMI, body mass index; NA, not available.

### 3.2 FM incidence in patients with OSAHS

**FIGURE 2:**
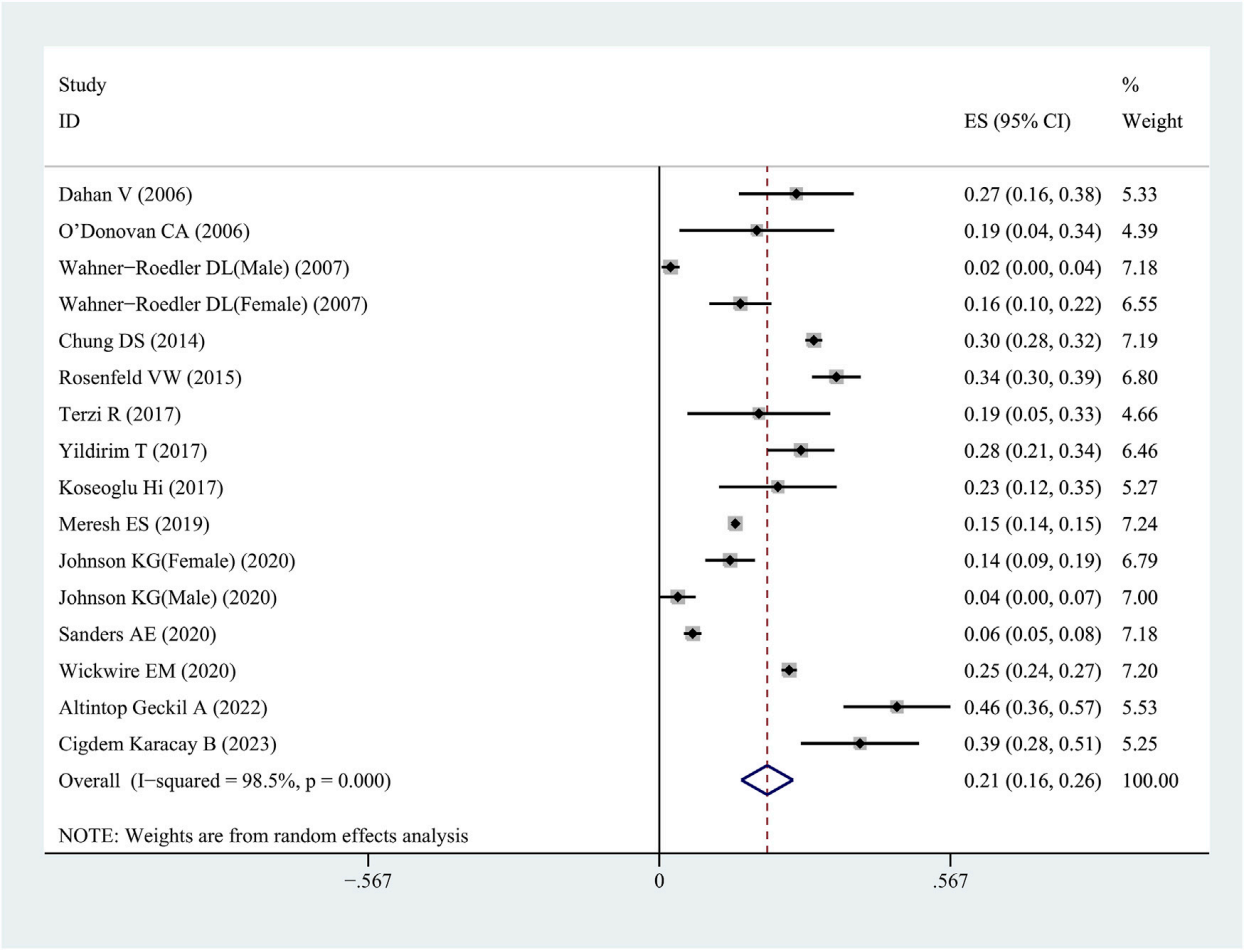
Forest plot of prevalence of Fibromyalgia in patients with OSAHS.

A meta-analysis of 14 publications from 16 studies was performed to examine the frequency of FM in patients with OSAHS (Table 1). A total of 23,473 patients were included in this study. Among these patients, 4,308 were tested positive and 19,169 negative for FM. The meta-analysis showed that the collective incidence of FM positivity was 21% (95% CI = 0.16–0.26, *p* < 0.001, I^2^ = 98.5%) after merging the effect values (Figure 2).

#### 3.2.1 Subgroup analysis

Males had an overall incidence of 3% of patients with OSAHS and positive FM (95% CI = 0.01–0.04, *p* = 0.002, I^2^ = 0%), meanwhile, females had an overall incidence of 23% (95% CI = 0.11–0.36, *p* < 0.001, I^2^ = 90.5%), according to a sex-based subgroup analysis (Table 2).

**TABLE 2:** Subgroup analyses of incidence of FM in OSAHS patients.

Subgroup analysis(n)	ES (95% CI)	*p*-value	I^2^ (%)	Ph
Overall (16)	0.21 (0.16–0.26)	<0.001	98.5	<0.001
Sex				
Male (2)	0.03 (0.01–0.04)	0.002	0	0.482
Female (4)	0.23 (0.11–0.36)	<0.001	90.5	<0.001
Mixed (10)	0.24 (0.18–0.30)	<0.001	98.5	<0.001
Ethnicity				
Caucasian (13)	0.21 (0.14–0.27)	<0.001	96.2	<0.001
Asian (1)	-	-	-	-
Mixed (2)	0.20 (0.10–0.30)	<0.001	99.4	<0.001
BMI				
BMI <30 (9)	0.18 (0.12–0.25)	<0.001	99.0	<0.001
BMI ≥30 (7)	0.25 (0.13–0.38)	<0.001	96.2	<0.001
Age				
Age <65 (14)	0.20 (0.15–0.26)	<0.001	98.4	<0.001
Age ≥65 (1)	-	-	-	-
Diagnostic criteria				
1990 ACR (4)	0.15 (0.03–0.27)	0.014	92.0	<0.001
2010 ACR (2)	0.32 (0.28–0.36)	<0.001	66.0	<0.001
2016 ACR (10)	0.20 (0.15–0.26)	<0.001	97.7	<0.001

Ph, P heterogeneity; ACR, american college of rheumatology.

According to racial-based subgroup analysis, the total incidence of patients in the Caucasian population with positive FM and OSAHS was 21% (95% CI = 0.14–0.27, *p* < 0.001, I^2^ = 96.2%) (Table 2); only one article (Chung et al., 2014) reported an overall incidence of 30% in Chinese.

BMI-based subgroup analysis revealed a 25% incidence of OSAHS and positive FM among patients with a mean BMI ≥30 (95% CI = 0.13–0.38, *p* < 0.001, I^2^ = 96.2%). Patients with a mean BMI <30 had an incidence of 18% OSAHS and positive FM (95% CI = 0.12–0.25, *p* < 0.001, I^2^ = 99.0%) (Table 2).

Only one publication (Wickwire et al., 2020) revealed a 25% incidence of OSAHS with positive FM among patients aged ≥65; OSAHS with positive FM was observed in 20% of patients aged <65 years (95% CI = 0.15–0.26, *p* < 0.001, I^2^ = 98.4%) (Table 2). Age was not specified sufficiently for classification in one study (Dahan et al., 2006).

Based on the criteria established in 1990, the proportion of patients with OSAHS and positive FM was 15% (95% CI = 0.03–0.27, *p* = 0.014, I^2^ = 92.0%); this figure increased to 32% (95% CI, 0.28–0.36, *p* < 0.001, I^2^ = 66.0%) and it reached 20% (95% CI = 0.15–0.26, *p* < 0.001, I^2^ = 97.0%) according to the 2016 criteria (Table 2).

#### 3.2.2 Meta-regression and sensitivity analyses

The incidence of FM in patients with OSAHS was shown to be very heterogeneous (I^2^ = 98.4%) in the combined analysis. Therefore, a meta-regression analysis was performed to determine the sources of heterogeneity. The results of the analysis indicated that the *p*-values for race, BMI, age, and sex were 0.078, 0.093, 0.114, and 0.063, respectively. These values indicate that the characteristics described above did not significantly influence heterogeneity. A sensitivity analysis was performed to remove 16 studies. The outcomes of the meta-analysis of the remaining articles were compared with those obtained before the initial exclusion. Eliminating individual studies did not have a significant impact on overall results. The sensitivity analysis of the incidence meta-analysis is shown in Supplementary Figure S1A. The incorporation of research into continuous variables for sleep monitoring indicators is limited. The study did not conduct a subgroup analysis or meta-regression.

#### 3.2.3 Publication bias

The meta-analysis of the incidence of FM did not show any indication of publication bias in the conjoint analysis using Egger’s (*p* = 0.191) and Begg’s (*p* = 0.152) methods (Supplementary Figure S1B).

### 3.3 Indicators of sleep monitoring that differ between patients with positive and negative FM

#### 3.3.1 AHI score

**FIGURE 3:**
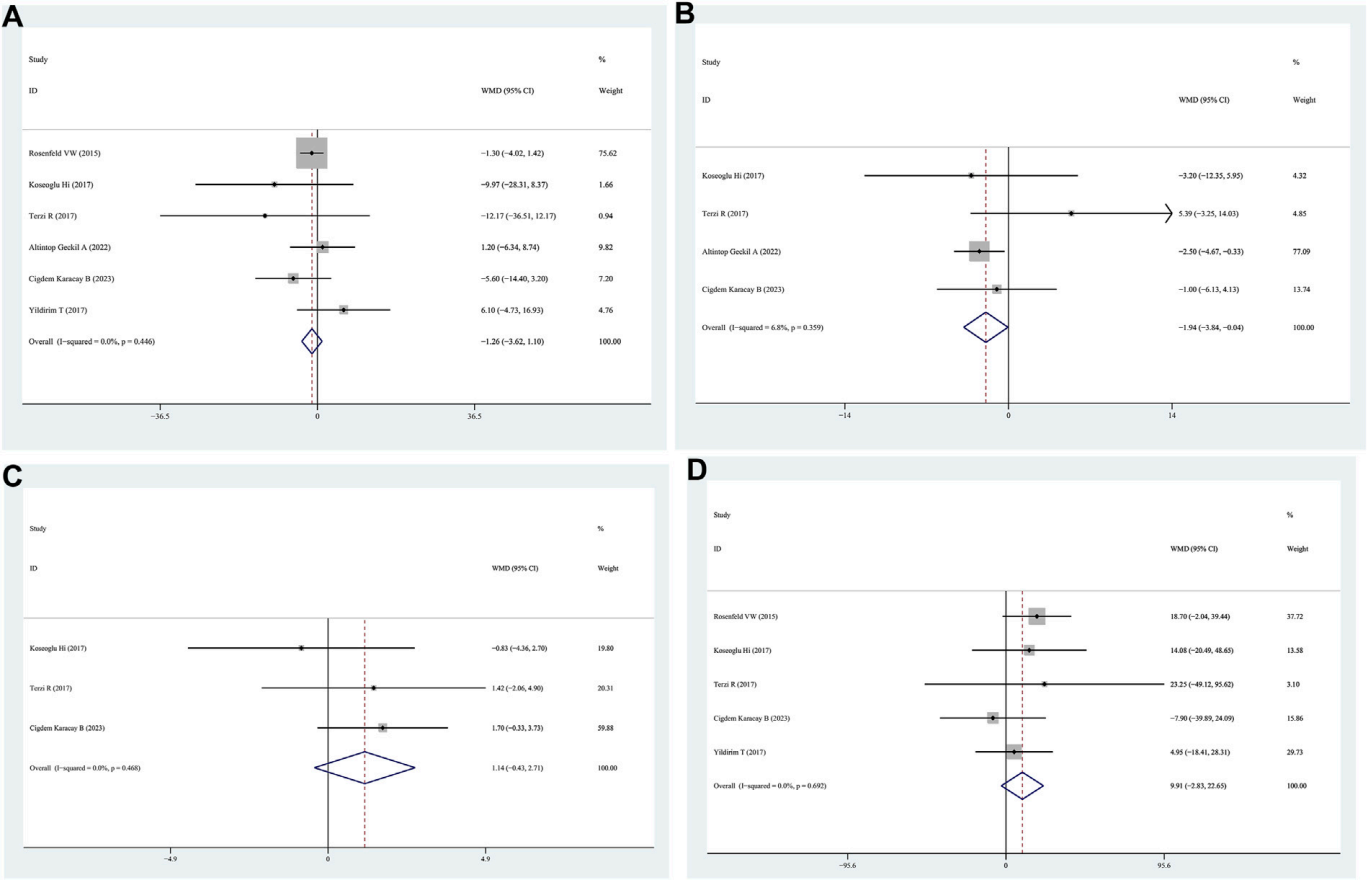
WMD forest plot and its 95%CI for AHI, Minimum SaO_2_, Mean SaO_2_, and total sleep time in FM + OSAHS group and FM- OSAHS group. **(A)** AHI; **(B)** Minimum SaO_2_; **(C)** Mean SaO_2_; **(D)** total sleep time.

Data on AHI scores of patients with positive FM compared to those with negative FM were obtained from six studies. The findings indicated that the AHI scores between the positive FM and negative FM groups were similar (WMD = −1.26, 95% CI = −3.62–1.10, *p* = 0.296, I^2^ = 0.0%) (Figure 3A).

#### 3.3.2 The minimum SaO_2_ level

Minimum SaO_2_ levels in patients with positive FM were compared to those in patients with negative FM in four studies. The patients in the positive FM group had a lower minimum SaO_2_ than those in the negative FM group, according to the findings (WMD = −1.94, 95% CI = −3.84–0.04, *p* = 0.045, I^2^ = 6.8%) (Figure 3B).

#### 3.3.3 The mean SaO_2_ level

Four studies reported lower SaO_2_ levels in patients with positive FM than in those with negative FM. The mean saturation oxygen levels of the positive FM and negative FM groups did not differ according to the data (WMD = 1.14, 95% CI = −0.43–2.71, *p* = 0.154, I^2^ = 0.0%) (Figure 3C).

#### 3.3.4 Total sleep time

Patients with positive FM were compared to those with negative FM in five studies that provided data on total sleep time. The findings indicated that there was no statistically significant distinction in the overall sleep time between patients in the positive FM group and those in the negative FM group (WMD = 9.91, 95% CI = −2.83–22.65, *p* = 0.128, I^2^ = 0%) (Figure 3D).

#### 3.3.5 Sleep latency

**FIGURE 4:**
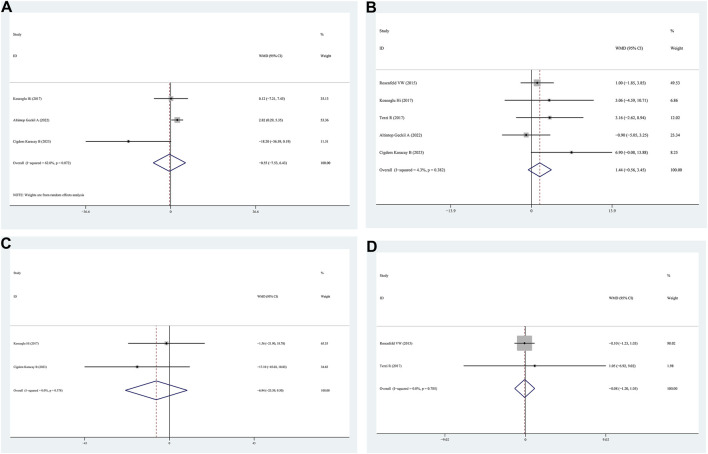
WMD forest plot and its 95%CI for Sleep latency, Sleep efficiency, REM, and Epworth Sleepiness Scale in FM + OSAHS group and FM- OSAHS group. **(A)** Sleep latency; **(B)** Sleep efficiency; **(C)** REM; **(D)** Epworth Sleepiness Scale. REM: rapid eye movement.

Sleep latency data were obtained from three studies comparing patients with positive and negative FM. The sleep latency values for the positive FM and negative FM groups did not differ according to the findings (WMD = −0.55, 95% CI = −7.53–6.43, *p* = 0.878, I^2^ = 62.0%) (Figure 4A).

#### 3.3.6 Sleep efficiency

Sleep efficiency data were obtained from five studies that compared patients with positive and negative FM. The findings indicated that the sleep efficiency values of the positive FM and negative FM groups were similar (WMD = 1.44, 95% CI = −0.56–3.45, *p* = 0.154, I^2^ = 4.3%) (Figure 4B).

#### 3.3.7 REM

REM data were obtained from two studies that compared patients with positive and negative FM. The findings (WMD = −6.94, 95% CI = −23.39–9.50, *p* = 0.408, I^2^ = 0.0%) indicated that there were no differences in REM between the positive FM and negative FM groups (Figure 4C).

#### 3.3.8 Epworth sleepiness scale

Data from two studies that compared patients with positive FM with those with negative FM on the Epworth sleepiness scale were presented. The findings indicated that no significant differences were observed between the positive FM and negative FM groups on the Epworth sleepiness scale (WMD = −0.08, 95% CI = −1.20–1.05, *p* = 0.893, I^2^ = 56.2%) (Figure 4D).

#### 3.3.9 N1/N2/N3

**FIGURE 5:**
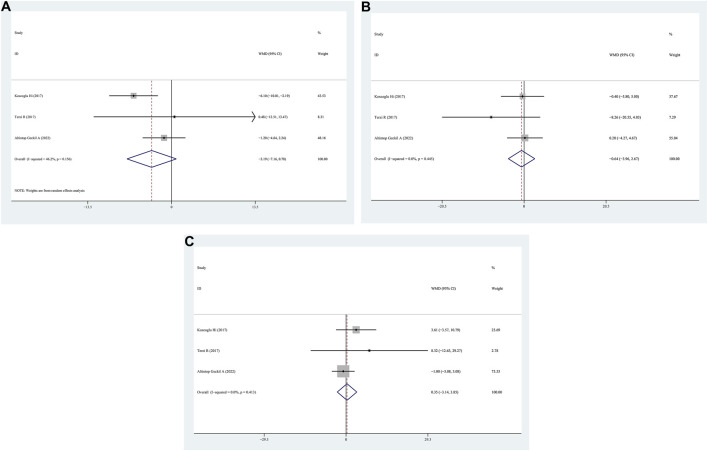
WMD forest plot and its 95%CI for N1/N2/N3 in FM + OSAHS group and FM- OSAHS group. **(A)** N1; **(B)** N2; **(C)** N3.

N1/N2/N3 data for patients with positive FM compared to those with negative FM were provided in three studies. No significant differences were observed in N1/N2/N3 between the positive FM and negative FM groups, according to the findings (WMD = −3.19, 95% CI = −7.16–0.78, *p* = 0.054, I^2^ = 56.2%; WMD = −0.64, 95% CI = −3.96–2.67, *p* = 0.704, I^2^ = 0.0%; N3: WMD = 0.35, 95% CI = −3.14–3.85, *p* = 0.844, I^2^ = 0.0%) (Figures 5A–C).

## 4 Discussion

This systematic evaluation and meta-analysis provide unique information on the clinical correlates of FM in patients with OSAHS. This study describes the incidence of FM in patients with OSAHS and the effects of race, BMI, age, and sex on the incidence of FM. Furthermore, this study compared differences in sleep monitoring indicators between the OSAHS with positive FM and OSAHS groups.

In general, the incidence of FM is high in patients with OSAHS (21%). Cigdem et al. (Cigdem Karacay et al., 2023) found that headache, afternoon fatigue, and morning fatigue were considerably more common in individuals with OSAHS and FM. In addition, patients with OSAHS and FM have low pain thresholds and high pain intensities. Patients with OSAHS may have more pressure points and a longer duration of chronic pain (Lahaye et al., 2023). Similarly, Terzi et al. (Terzi and Yılmaz, 2017) discovered that patients with OSAHS and FM had lower total myalgic and control scores than patients with only OSAHS. Intermittent hypoxia increases pain sensitivity by altering the pain transmission pathway in patients with OSAHS (Wu et al., 2015). Therefore, patients with OSAHS have a higher incidence of FM. In particular, a higher percentage of female patients with OSAHS had comorbid FM than male patients with OSAHS. Different diagnostic criteria affected the incidence of FM, which was supported by subgroup analysis based on diagnostic criteria in this study. An epidemiological survey based on the 1990 ACR of rheumatology diagnostic criteria showed a male-to-female ratio of 9:1 for FM, which was attributed to the fact that a significant number of male patients with peripheral pain had unremarkable somatic tenderness (Jiang et al., 2020). In contrast, the diagnostic criteria for FM revised by the ACR in 2010 and 2016 do not require a diagnosis based on the number of pressure points, revealing more male patients with FM and a 3:1 female-to-male ratio (Queiroz, 2013). However, its incidence remains higher in women than in men. Most previous studies have shown that women experience more pressure points than men (Jiao et al., 2021). Females have lower pain thresholds and show a higher sensitivity to noxious stimuli from mechanical stress and electrical, thermal, ischemic, and cold stimuli, which may be related to differences in sex hormones, endogenous analgesic systems, brain structure, function, sex chromosomes, and psychosocial factors between women and men (Fillingim et al., 2009; Mogil, 2012; Bartley and Fillingim, 2013; Packiasabapathy and Sadhasivam, 2018; Meyer-Frießem et al., 2020; Sauch et al., 2022).

In addition, the incidence of FM in patients with OSAHS varies by race, which could be attributed to variations in genetic polymorphisms, lifestyle habits, and body size in populations from different regions (Zorina-Lichtenwalter et al., 2024). Furthermore, the incidence of FM was slightly higher in obese patients with OSAHS. There is a bidirectional association between OSAHS and obesity (He et al., 2022), which is a critical factor that influences FM (D'Onghia et al., 2021). One study suggested that obese females with FM are more likely to experience chronic pain than non-obese females with FM (Koçyiğit and Okyay, 2018). Obesity leads to a higher prevalence of OSAHS and FM, probably due to joint pain caused by joint loading due to high BMI and generalized pain due to thick subcutaneous fat (Costa et al., 2023; Mathkhor and Ibraheem, 2023). Furthermore, obesity aggravates the severity of OSAHS, and shortening of sleep duration and poor sleep quality promote pain sensitivity (Larsen et al., 2022).

This study found a 22% incidence of FM in patients aged <65 years. Wickwire et al. (Wickwire et al., 2020) reported that the overall incidence of FM in older patients with OSAHS and positive FM was 25%. A previous study showed that the duration of FM symptoms increases with age (Cronan et al., 2002). Sarzi-Puttini (Sarzi-Puttini et al., 2020) reported that FM is the third most common musculoskeletal disorder, and its incidence increases with age. This could be explained by the fact that the organism is more susceptible to pain and fatigue with age, which are closely related to aging, and older people may suffer from other age-related health problems that exacerbate their symptoms of FM.

In particular, the patient had depression, anxiety, restless legs syndrome, elevated autoimmune titers, migraine, and possible vulvodynia or vaginismus as comorbidities of FM (Treves and Qazi, 2022). Stehlik et al. (Stehlik et al., 2009) found that 64% of a group of female patients diagnosed with FM also concurrently suffered from restless legs syndrome. Compared to patients who suffer only from FM, patients with both FM and restless legs syndrome experience sleep disturbances and pronounced daytime sleepiness more frequently. Civelek et al. (Civelek et al., 2014) also reported that the prevalence of restless legs syndrome was higher in the FM group than in the normal population, and the quality of sleep and quality of life were worse in patients with restless legs syndrome. We contend that patients with OSAHS and restless leg syndrome may be more prone to FM. Consistent with these findings, in the study by Altıntop et al., periodic limb movements in sleep (PLMS) was found to be significantly higher in the OSAHS with positive FM group than in the OSAHS with negative FM group (Altıntop Geçkil and Aydoğan Baykara, 2022). One study (Tayag-Kier et al., 2000) suggested that juvenile FM subjects exhibit excessive movement activity during sleep. A total of 38% of patients with FM had an abnormally elevated PLMS index (>5/h), indicating the presence of PLMS in these subjects (Tayag-Kier et al., 2000). The abovementioned studies imply that restless legs syndrome and PLMS might be confounding factors when evaluating the incidence of FM in patients with OSAHS. Unfortunately, the included studies lacked data on possible confounding factors, such as the number of individuals with restless legs syndrome and PLMS; therefore, we were unable to adjust for these factors. We hope that in the future, more studies will focus on the detection of restless legs syndrome and PLMS in these populations (FM plus OSAHS).

It is characterized by chronic generalized discomfort, fatigue, and sleep difficulties (López-Medina and Moltó, 2020). FM is associated with comorbidities such as rheumatism, mental disorders, gastrointestinal disorders, cardiovascular disorders, and peripheral neuropathy (Muzammil and Cooper, 2011; Duan et al., 2019; Wu et al., 2021). Sleep difficulties are common in patients with FM (Lee et al., 2016). Patients with FM have a short duration of sleep, resulting in poor sleep, poorer sleep quality, and impaired sleep efficiency (Martinsen et al., 2014). Rizzi et al. (Rizzi et al., 2017) showed that sleep had the same effects as stress in patients with FM. A vicious circle is created during sleep: pain increases sympathetic cardiovascular activation and reduces sleep efficiency, causing lighter sleep, higher cyclic alternating pattern rate, more arousal, higher PLMS index, and an increased occurrence of periodic breathing, leading to abnormal cardiovascular neural control and exaggerated pain sensitivity. Aberrant autonomic nervous system responses are biological markers of FM (Kang et al., 2016). Patients with FM show sympathetic and parasympathetic hypoactivity in autonomic function (Martínez-Lavín et al., 1998; Raj et al., 2000; Cohen et al., 2001). In FM, sympathetic vascular modulation is reduced, cardiac vagal withdrawal is impaired, and orthostatic tolerance is reduced (Furlan et al., 2005). Autonomic dysfunction is characterized by sustained sympathetic hyperactivity and hyperreactivity to stress in autonomic dysfunction (Di Franco et al., 2010). One study reported that the blunted heart rate response during exercise observed in patients with FM could be associated with the desensitization of cardiac β1 receptors through a heightened sympathetic activity similar to heart failure (Lauer, 2004). Therefore, autonomic cardiovascular dysfunction may be involved in the complex etiopathogenesis of FM syndrome and increase the risk of cardiac events.

Sleep plays an important role in the etiology and treatment of FM. Alpha-wave intrusion during deep sleep is a common sleep disturbance associated with FM (Roizenblatt et al., 2011). Sleep-disordered breathing has also been associated with FM in some studies (Shah et al., 2006). Women with FM experience disordered breathing (Shah et al., 2006). FM may be a marker of occult sleep apnea in males (May et al., 1993). This is a particularly notable correlation to explore because care for patients with FM is often scattered due to comorbidities (Garcia-Campayo et al., 2008; Munipalli et al., 2022). As patients with OSAHS and FM commonly suffer from psychiatric comorbidities, they are often treated by psychiatric and pain clinics that prescribe benzodiazepines and opioids (Thorpe et al., 2018; Wang et al., 2019; Cutrufello et al., 2020). Opioids combined with benzodiazepines can worsen OSA outcomes and patients may become dependent on opioid painkillers (Fitzcharles et al., 2011; Cunningham et al., 2016). The mechanisms by which the brain modulates pain involve complex pathways, the neural circuits underlying which are poorly understood. Hyperalgesia is associated with a loss of 4 h of REM sleep (Roehrs et al., 2006). Rosenfeld et al. (Rosenfeld et al., 2015) reported a unique electroencephalography in patients with FM and reported that the incidence of complications associated with OSAHS was 45%. They also found a low delta/alpha ratio during non-REM sleep in patients with FM. Some studies have shown increased sympathetic activity during sleep in both OSAHS and FM (Kang et al., 2016; Tang et al., 2022), and that the pathophysiological mechanisms of both disorders involve central sensitization and serotonin deficiency (Altıntop Geçkil and Aydoğan Baykara, 2022). In addition, some clinical manifestations of FM, such as fatigue, reduced exercise capacity, and cold intolerance, can be explained by growth hormone deficiency. Moreover, the levels decrease further as FM progresses (Jones et al., 2007). Approximately 70% of the daily release of growth hormone occurs during the N3 and REM sleep phases (Van Cauter et al., 1998). OSAHS is a significant factor that affects the GH release of growth hormone (Francisco et al., 2023). Therefore, FM and OSAHS share common pathological mechanisms and the two conditions mutually influence each other, creating a vicious cycle.

FM can be managed more effectively using a multidisciplinary strategy that includes rheumatologists, physiotherapists, and psychiatrists. Some studies have recommended polysomnographic monitoring, particularly in patients with FM with severe daytime drowsiness (Mutlu et al., 2020). Patients with FM can benefit from OSAHS evaluation and patients with OSAHS may benefit from the FM assessment (Cappelleri et al., 2009; Köseoğlu et al., 2017). In this study, most of the sleep monitoring indicators did not differ substantially between patients with OSAHS with positive FM and those with OSAHS with negative FM. However, when patients with OSAHS experience chronic pain, such as FM, it is crucial to monitor changes in pain thresholds. Furthermore, the effects of nocturnal hypoxemia and sleep disturbances on pain thresholds were evaluated. Recent findings contradict those of previous studies on the association between OSAHS severity and FM coexistence. Koseoglu et al. (Köseoğlu et al., 2017) demonstrated that FM and OSAHS did not affect polysomnographic indicators. However, this meta-analysis showed that patients with OSAHS and FM had a lower minimum oxygen saturation, suggesting muscle dysfunction due to increased tissue hypoxia, and this may explain the increased pain complaints in patients with FM. However, in terms of several sleep monitoring metrics, including AHI score, sleep latency, mean saturation oxygen level, sleep efficiency, total sleep duration, N1/N2/N3, REM, and the Epworth sleepiness scale score, the OSAHS with positive FM group did not differ significantly from the OSAHS with negative FM group. However, a link between AHI and FM severity has previously been documented previously (Mutlu et al., 2020). Altıntop et al. (Altıntop Geçkil and Aydoğan Baykara, 2022) reported longer sleep latency in patients with OSAHS and FM. These findings indicate a lack of consensus on the association between sleep monitoring indices in OSAHS and FM, which could be attributed to the small sample size of a single study. Second, different environments and equipment for sleep monitoring affected the data; therefore, larger clinical studies are needed. In summary, this study suggests that patients with OSAHS have a high incidence of FM, which may help clinicians assess FM in patients with OSAHS and implement proactive interventions. Continuous positive airway pressure (CPAP) therapy is the main treatment for OSAHS (Gagnadoux et al., 2011). In a study that evaluated 14 patients with OSAHS, there was a significant improvement in FM symptoms after 3 weeks of CPAP therapy (Gold et al., 2004). Furthermore, opioids and benzodiazepines used to treat FM can exacerbate OSAHS and increase pain (Cunningham et al., 2016). Therefore, determining the incidence and performing early treatments such as CPAP in patients with OSAHS and FM can prevent unnecessary FM treatment.

This study has several limitations. First, the heterogeneity may have been caused by differences in the definition of FM between studies, subject characteristics, and diagnostic thresholds. Therefore, interpreting meta-analysis data is difficult. Second, due to the lack of data, this review was unable to establish a causal link between OSAHS and FM. Third, these studies lack concrete evidence to highlight the clinical impact of FM on healthcare expenditure, an issue that future research should examine. Fourth, differences in severity between FM and pain measurement standards can restrict the therapeutic applicability of the data. The difficulty in diagnosing FM and the successive definitions of this pathology over time certainly limit the scope of the results (Qureshi et al., 2021). The latest definition is more rigorous and the literature published before 2015 should be treated with caution.

## 5 Conclusion

The incidence of FM is high among patients with OSAHS. In clinical practice, patients with OSAHS undergo FM screening. Additionally, most of the sleep monitoring indicators in patients with OSAHS and FM were not significantly different from those in patients with OSAHS but without FM.

## Data Availability

The original contributions presented in the study are included in the article/Supplementary Material, further inquiries can be directed to the corresponding authors.
