# Supplementary material for: Fibromyalgia in obstructive sleep apnea-hypopnea syndrome: a systematic review and meta-analysis
Source: Front Physiol. 2024 May 20;15:1394865. doi: 10.3389/fphys.2024.1394865 (PMC11144865; doi:10.3389/fphys.2024.1394865)
Supplement: Supplementary file 1 [file Table1.DOCX]

| Author |  | Rapid Eye Movement | |  | N1 | |  | N2 | |  | N3 | |  | Epworth Sleepiness Scale | |
| --- | --- | --- | --- | --- | --- | --- | --- | --- | --- | --- | --- | --- | --- | --- | --- |
|  |  | FM+ | FM- |  | FM+ | FM- |  | FM+ | FM- |  | FM+ | FM- |  | FM+ | FM- |
| Rosenfeld VW |  |  |  |  |  |  |  |  |  |  |  |  |  | 10.40±5.40 | 10.50±5.40 |
| Koseoglu Hi |  | 62.67±31.93 | 64.23±30.15 |  | 10.95±5.71 | 17.05±7.12 |  | 41.22±7.53 | 41.62±10.73 |  | 31.73±10.51 | 28.12±12.99 |  |  |  |
| Terzi R |  |  |  |  | 11.10±15.28 | 10.62±11.17 |  | 16.00±13.12 | 24.26±16.28 |  | 48.35±24.66 | 40.03±17.93 |  | 10.50±9.68 | 9.45±4.83 |
| Altintop Geckil A |  |  |  |  | 8.70±9.60 | 9.90±14.40 |  | 54.00±16.00 | 53.80±15.30 |  | 23.90±14.20 | 24.90±14.40 |  |  |  |
| Cigdem Karacay B |  | 32.30±27.20 | 49.40±85.90 |  |  |  |  |  |  |  |  |  |  |  |  |
| Yildirim T |  |  |  |  |  |  |  |  |  |  |  |  |  |  |  |

Supplementary table 1 sleep indexes for FM+ and FM- patient group.
