# Supplementary figures and images for: Fibromyalgia in obstructive sleep apnea-hypopnea syndrome: a systematic review and meta-analysis
Source: Front Physiol. 2024 May 20;15:1394865. doi: 10.3389/fphys.2024.1394865 (PMC11144865; doi:10.3389/fphys.2024.1394865)

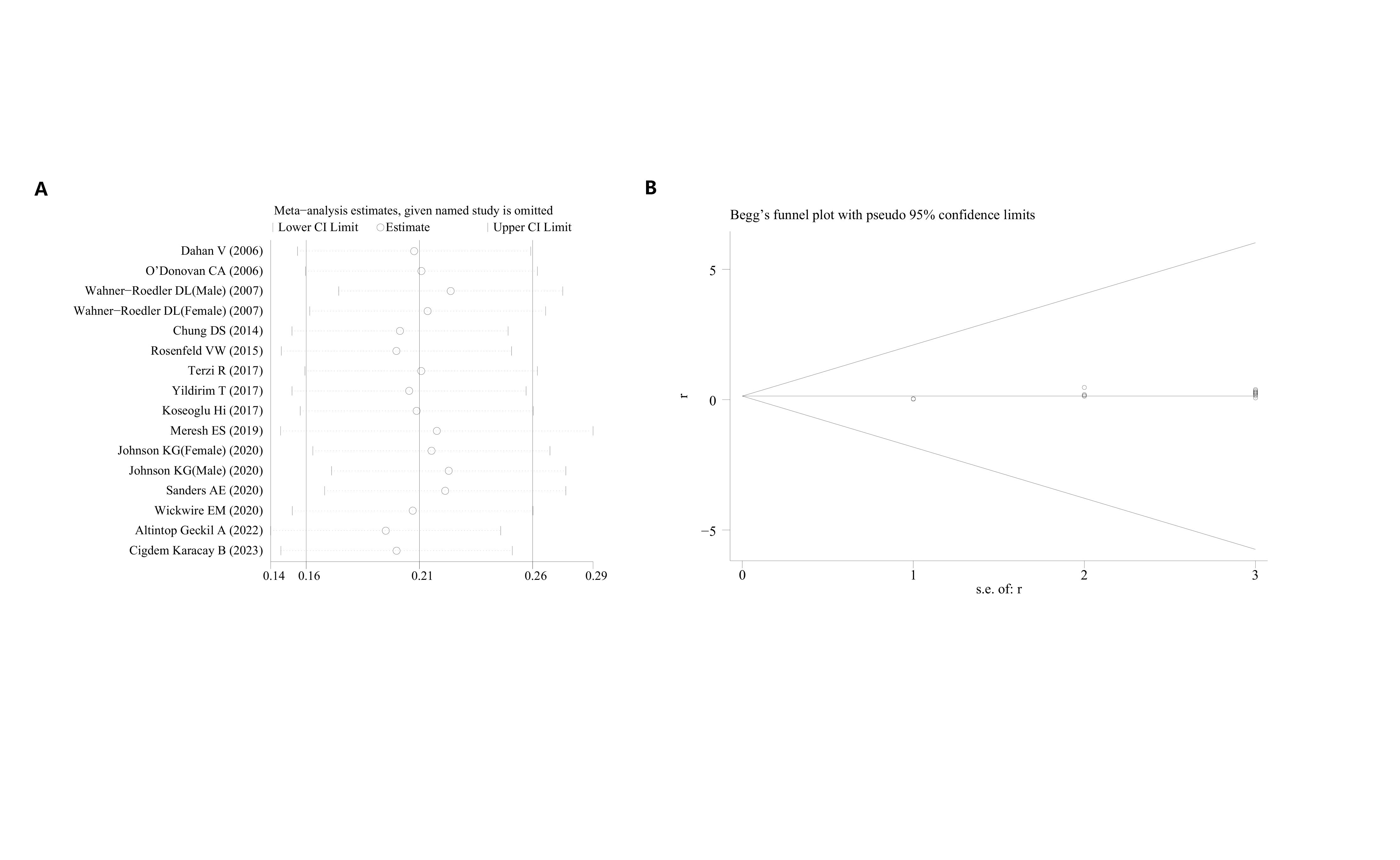

Supplement: Supplementary file 2 [file Image1.TIF]
