# Supplementary material for: Fibromyalgia in obstructive sleep apnea-hypopnea syndrome: a systematic review and meta-analysis
Source: Front Physiol. 2024 May 20;15:1394865. doi: 10.3389/fphys.2024.1394865 (PMC11144865; doi:10.3389/fphys.2024.1394865)
Supplement: Supplementary file 3 [file Table2.DOCX]

|  |  | |  |  | |  |  | |  |  | |  |  | |  |  | |
| --- | --- | --- | --- | --- | --- | --- | --- | --- | --- | --- | --- | --- | --- | --- | --- | --- | --- |
| Author | AHI | |  | Minimum SaO2 | |  | Mean saturation oxygen | |  | Total sleep time | |  | Sleep latency | |  | Sleep efficiency | |
|  | FM+ | FM- |  | FM+ | FM- |  | FM+ | FM- |  | FM+ | FM- |  | FM+ | FM- |  | FM+ | FM- |
| Rosenfeld VW | 9.40±14.80 | 10.70±8.30 |  |  |  |  |  |  |  | 304.60±95.80 | 285.90±104.1 | |  |  |  | 78.50±12.60 | 77.50±15.20 |
| Koseoglu Hi | 33.86±28.93 | 43.83±26.68 |  | 73.25±14.39 | 76.45±13.48 |  | 91.42±5.82 | 92.25±4.07 |  | 377.42±53.65 | 363.34±53.35 | | 15.00±11.66 | 14.88±10.32 |  | 82.13±12.10 | 79.07±11.03 |
| Terzi R | 24.55±26.45 | 36.72±30.65 |  | 82.83±9.28 | 77.44±11.29 |  | 94.08±3.37 | 92.66±5.62 |  | 434.25±81.97 | 411.00±78.03 | |  |  |  | 88.18±4.90 | 85.02±10.83 |
| Altintop Geckil A | 39.20±24.60 | 38.00±28.40 |  | 78.30±7.60 | 80.80±7.60 |  |  |  |  |  |  |  | 16.09±9.80 | 13.27±7.70 |  | 75.30±15.90 | 76.20±12.80 |
| Cigdem Karacay B | 30.80±17.10 | 36.40±19.80 |  | 79.00±11.00 | 80.00±10.00 |  | 93.20±2.40 | 91.50±6.00 |  | 380.80±65.30 | 388.70±67.50 | | 24.40±31.80 | 42.60±46.10 |  | 79.50±10.00 | 72.60±19.40 |
| Yildirim T | 39.90±34.80 | 33.80±28.70 |  |  |  |  |  |  |  | 358.65±69.88 | 353.70±76.29 | |  |  |  |  |  |

Supplementary table 2 sleep indexes for FM+ and FM- patient group.
